# Factors influencing the choice of facility-based delivery in the ethnic minority villages of Lao PDR: a qualitative case study

**DOI:** 10.1186/s41182-019-0177-2

**Published:** 2019-09-05

**Authors:** Chika Sato, Khampheng Phongluxa, Noriko Toyama, Ernesto R. Gregorio, Chiaki Miyoshi, Futoshi Nishimoto, Tomomi Takayama, Tiengkham Pongvongsa, Kenzo Takahashi, Sengchanh Kounnavong, Jun Kobayashi

**Affiliations:** 1 Asia Health and Education Fund, Tokyo, Japan; 2 Department of Global Health, Graduate School of Health Sciences, University of the Ryukyus, Okinawa, Japan; 3 Lao Tropical and Public Health Institute, Ministry of Health, Vientiane, Lao People’s Democratic Republic; 4 Department of Community Nursing, School of Health Sciences, Faculty of Medicine, University of the Ryukyus, Okinawa, Japan; 5 Department of Health Promotion and Education, College of Public Health, University of the Philippines, Manila, Philippines; 6 SEAMEO-TROPMED Regional Center for Public Health, Hospital Administration, Environmental & Occupational Health, Manila, Philippines; 7 Bureau of International Health Cooperation, National Center for Global Health and Medicine, Tokyo, Japan; 8 Nagasaki University School of Tropical Medicine and Global Health, Nagasaki, Japan; 9 Savannakhet Provincial Health Department, Savannakhet, Lao People’s Democratic Republic; 10 Teikyo University Graduate School of Public Health, Tokyo, Japan

**Keywords:** Facility-based delivery, Decision-making, Ethnic minority, Qualitative case study, Thematic analysis

## Abstract

**Background:**

Facility-based delivery has been promoted to improve maternal and child health care in Lao PDR and a free delivery policy was introduced at designated health care facilities (HCF) in 2013. However, according to birth records of HCFs in the impoverished and remote district, only a few women utilized the HCFs despite good physical accessibility. The aim of this study was to analyze the factors influencing the choice of facility-based delivery in the impoverished and remote district after a free delivery policy was introduced.

**Methods:**

Qualitative case study was employed. Focus group discussions (FGDs) and in-depth interviews (IDIs) were conducted from August to October 2015. Five hamlets (or small village) located along the main road where only a few women delivered at HCFs were selected for the study based on birth records. The participants of the FGDs and IDIs were the village heads, village health volunteers, women who delivered at home or at a health facility within the past 2 years, their husbands, and mothers or mothers-in-law. Thematic analysis was used to analyze the data.

**Results:**

A total of 12 FGDs and 27 IDIs were conducted, and the number of participants was 105. The factors influencing the choice of facility-based delivery were classified into nine categories and 19 subcategories. The categories were labeled, “perception of childbirth,” “traditional health concept: *sabaai* (a condition of health, ease, and comfort),” “perception of health care facilities and staff,” “previous pregnancy and childbirth experience,” “mode of available transportation,” “financial burden of childbirth at health care facility,” “family and community context,” “institutional context,” and “government policy on delivery.”

**Conclusion:**

Our study demonstrated that five major factors negatively influenced the choice of facility-based delivery: (1) perception of childbirth, (2) preference for *sabaai*, (3) financial burden, (4) family decision-making, and (5) institutional context. To promote facility-based delivery in the impoverished and remote district, three strategies are recommended: (1) promoting community-based health education involving women and strengthening community-based mutual support, (2) clarifying items essential for delivery at HCFs, and (3) making HCFs more comfortable in terms of “*sabaai*.”

## Background

The Lao People’s Democratic Republic (Lao PDR) is a low-income country located between Thailand and Vietnam with a total population of 6.8 million. It is a multiracial nation with 49 ethnic groups, the largest of which are the Lao, who make up 53.2% of the total population [1, 2].

The Ministry of Health of Lao PDR has promoted facility-based delivery as a means of improving maternal and child health and introduced a free delivery policy at designated health care facilities (HCF) in 2013 [3]. All public health facilities are designated health facilities in Lao PDR with at least one skilled birth attendant dispatched in all public health facilities (health center, district and provincial hospital) to promote free delivery. Most maternal deaths occurred during and within 24 h of delivery [4]. Home-based deliveries have a higher risk of maternal death due to the lack of qualified emergency care in the community and the difficulty of transporting expectant mothers to a HCF, the principal reason for Campbell’s recommendation of facility-based delivery [5].

The maternal mortality ratio (MMR), defined as the number of maternal deaths per 100,000 live births, decreased from 905 in 1990 to 197 in 2015 [6]. Other maternal and child health indicators such as the infant mortality rate (IMR) and under 5 mortality rate (U5MR) also decreased [7]. The percentage of facility-based delivery in Lao PDR increased from 17.1% in 2005 to 37.5% in 2012 [8, 9]. However, the difference in the percentages of facility-based delivery between the urban (74.2%) and rural areas (27%) remained large [9].

Bohren in 2014 reviewed the facilitators and barriers to facility-based delivery in low- and middle-income countries [10]. Thirty-four studies from 17 countries in Africa (8 countries), Asia (7 countries), and South America (1 country) were reviewed. The findings were organized under four broad themes: (1) perceptions of pregnancy and childbirth, (2) influence of socio-cultural context and care experiences, (3) resource availability and access, and (4) perceptions of quality of care. Another study which explored why rural Laotians in Khammouane and Champasack province of Lao PDR chose home deliveries in 2008–2009 demonstrated the influence of three factors: (1) perceived advantages of giving birth at home, such as ease, convenience, nearness to family, traditional postpartum practices, and previous positive experiences; (2) perceived disadvantages of giving birth in a HCF such as cost, transport, and previous negative experiences; and (3) decision-making by family regarding the place of delivery, such as the role of the husband or other family members as the primary decision maker and the influence of the mother or mother-in-law [11]. According to the results of a previous study, the key barriers to facility-based delivery in Lao PDR were identical with those found in other countries.

Xepon district, one of 47 priority districts targeted by the government for poverty relief, is located in the central Laotian province of Savannakhet. Most of the residents belong to an ethnic minority. According to estimates by Nishimoto [12], the IMR of this district in 2013 was 69.8 per 1000 live births while the U5MR was 95.6 per 1000 live births; both were higher than the national average. The IMR by place of delivery was 139 at home and 34 at HCF [13]. Even though home-based delivery posed a high risk for infants, very few women in Xepon chose to deliver at a HCF. Despite this fact, there has been no study that addressed the factors influencing the choice of place of delivery after a free delivery policy was introduced in Xepon district.

The aim of this study was therefore to analyze the factors influencing the choice of facility-based delivery and to recommend effective interventions designed to promote HCF as a place for delivery in the impoverished and remote district in Lao PDR after a free delivery policy was introduced.

## Method

### Study design

This study was a qualitative case study design and used focus group discussions (FGDs) and in-depth interviews (IDIs) for data collection.

### Study site

Xepon district, one of 47 priority districts targeted by the government for poverty relief, is located in the central Laotian province of Savannakhet. Five hamlets (or small village) located along the main road where only a few women delivered at health care facilities were selected for this study based on the birth records at the facilities. The distance from these hamlets to the local health care facility was 4–8 km, while the distance to the district hospital was 17–21 km. There was no traditional birth attendant in any of these hamlets.

### Participants

The participants of the FGDs were women who delivered at home within the past 2 years, their husband, and mother or mother-in-law. The participants of the IDIs were village heads, village health volunteers, women who delivered at home or at a HCF within the past 2 years, their husband, and mother or mother-in-law. Participants were purposively selected by the village head or village health volunteers. There was no list of women who delivered within the past 2 years in the remote and rural villages, and foreigners could not select participants randomly under the present political system in Lao PDR. For women who had given birth more than twice, the last place of delivery was selected for the study. Women who were less than 16 years old were excluded from the study.

### Research instrumentation

FGDs and IDIs were conducted by trained Laotians using a Laotian translation of an English-language interview manual. The semi-structured interview guide was used in both the FGDs and IDIs. The interview guide included questions regarding the place of delivery, the person deciding the place of delivery, the reasons for choosing the place of delivery, the person(s) attending the delivery, the kinds of support received from health facilities, and problems encountered during the pregnancy and delivery. A pilot test was conducted in a rural area of Vientiane, and the needed revisions were made to the interview manual and procedure.

### Data collection

Five Laotian health staff were purposively selected from the National Institute of Public Health and Savannakhet provincial health office and trained as interviewers. In cases where the participants experienced difficulty understanding Laotian, villagers who speak both Laotian and the ethnic minority language helped during the interviews

FGDs were conducted by two moderators, one note-taker, and one observer. The FGDs were conducted at the home of the village head, and the IDIs were conducted either at the home of village head or of the participants after the introduction and informed consent. Each FGD lasted from 40 to 60 min, while each IDI lasted from 30 to 40 min. With permission from the participants, both FGDs and IDIs were recorded using a digital voice recorder. Sociodemographic characteristics of the participants were collected before FGDs and IDIs using face sheet of interview guide.

Afterwards, the transcripts of the FGDs and IDIs were translated into Lao by native Laotians who understood the study objectives, then translated into English by a Laotian proficient in the language and also knew the study objectives.

### Data analysis

The sociodemographic variables were analyzed and presented as frequencies and proportions while qualitative data was analyzed using thematic analysis. The thematic analysis was used to create codes and categories. First, words or phrases related to a theme, such as the factors influencing the choice of facility-based delivery, were selected from the field notes and transcripts of the FGDs and IDIs. Second, related data were grouped to create categories. Third, the categories were structured based on a comparison of the content between categories and on the association of theme to content. Repeated restructuring was conducted.

“Strategies for establishing reliability and validity in qualitative research” by Holloway and Wheeler was used to ensure reliability and validity [14]. We followed a strategy consisting of member check, peer review, triangulation, audit or decision trail, thick description, and reflexivity. A peer review was conducted through committee meetings with professional Laotian educators and experts in child and maternal health. Furthermore, this study was supervised by an expert in qualitative research in public health.

## Results

### Characteristics of study participants

A total of 12 FGDs and 27 IDIs were conducted. There were a total of 105 participants consisting of five village heads, three village health volunteers, 29 women who delivered at home, 26 husbands, 28 mothers or mothers-in-law, ten women who delivered at a health facility, and four husbands.

Table 1 shows the characteristics of all of the participants. Participants’ age ranged from 17 to 72 years (median age 33). In terms of ethnicity, 74.3% of the participants were Tree and 21% were Makong. The majority of participants (71.4%) had no formal education, 22.9% had a primary school education (1–5 years), 4.8% had a secondary school education (6–11 years), and only 1% had more than a high school education. All but one participant, a teacher, were farmers. Most ethnic Laotians are Buddhist, but 92.4% of the participants in the present study were Animists.

**Table 1:** Socio-demographic characteristics of the study participants

	Total (*n* = 105)	Women who gave birth (*n* = 39)	Husbands (*n* = 30)	Mothers/mothers-in-law (*n* = 28)	Village heads/VHVs (*n* = 8)
Age: median (range)	33 (17–72)	27 (17–43)	31.5 (18–47)	49 (40–72)	40.5 (29–57)
Ethnic group: *n* (%)					
Tree	78 (74.3)	27 (69.2)	24 (80.0)	22 (78.6)	5 (62.5)
Makong	22 (21.0)	10 (25.6)	5 (16.7)	6 (21.4)	1 (12.5)
Phoutha	3 (2.9)	1 (2.6)	0 (0.0)	0 (0.0)	2 (25.2)
Other	2 (1.9)	1 (2.6)	1 (3.3)	0 (0.0)	0 (0.0)
Education: *n* (%)					
0 years	75 (71.4)	33 (84.6)	14 (46.7)	28 (100.0)	0 (0.0)
1–5 years	24 (22.9)	5 (12.8)	13 (43.3)	0 (0.0)	6 (75.0)
6–11 years	5 (4.8)	0 (0.0)	3 (10.0)	0 (0.0)	2 (25.0)
More than 12 years	1 (1.0)	1 (2.6)	0 (0.0)	0 (0.0)	0 (0.0)
Religion: *n* (%)					
Animism	97 (92.4)	37 (94.9)	27 (90.0)	26 (92.9)	7 (87.5)
Buddhism	3 (2.9)	2 (5.1)	1 (3.3)	2 (7.1)	0 (0.0)
No answer	5 (4.8)	0 (0.0)	2 (6.7)	0 (0.0)	1 (12.5)

### Factors influencing choice of delivery place

Table 2 shows the categorized results of the thematic analysis. The factors influencing the choice of facility-based delivery were classified into nine categories and 19 subcategories. The categories were labeled: “perception of childbirth,” “traditional health concept: *sabaai* (a condition of health, ease, and comfort),” “perceptions of health care facilities and staff,” “previous pregnancy and childbirth experience,” “mode of available transportation,” “financial burden of childbirth at health care facility,” “family and community context,” “institutional context,” and “government policy on delivery.” Each category consisted of one or multiple subcategories as described in Table 2.

**Table 2:** Factors influencing choice of facility-based delivery

Category	Subcategory
Perceptions of childbirth	Childbirth as a natural event
	Desire for medical treatment due to fear about child mortality and difficulties during childbirth
	Religious beliefs about childbirth
Traditional health concept: “*sabaai*”	*Sabaai* as healthy
	*Sabaai* as easy
	*Sabaai* as comfortable
Perceptions of health care facilities and staff	Care at health care facilities
	Service from health care staff
Previous pregnancy and childbirth experience	Previous childbirth experience
	Experience of ANC
Mode of available transportation	Transportation to health care facility
Financial burden of childbirth at health care facility	Cost of transportation in going to a health care facility
	Indirect cost of childbirth at a health care facility
	Hidden cost of childbirth at a health care facility
Family and community context	Personal links with others
	Decision-making in the family
Institutional context	Difficulty preparing for a facility-based delivery
	Language difficulties
Government policy on delivery	Free delivery policy at health care facilities

#### Perceptions of childbirth

“Perceptions of childbirth” consisted of three subcategories including “childbirth as a natural event,” “desire for medical treatment due to fear about possible child mortality and difficulties during childbirth,” and “religious beliefs about childbirth.”

##### Childbirth as a natural event

Participants recognized childbirth as a natural event rather than as a disease and therefore did not require antenatal care (ANC) or delivery at a HCF. One participant said:

“Every village does the same if the delivery is not difficult. Women deliver at home, and we do not want health staff to assist if the delivery is easy.” (Husband)

Participants were accustomed to the idea of delivery at home rather than at a facility. These comments were made by mothers and mothers-in-law because home-based delivery was more common for their generation. One participant said:

“The mother was the person who decided on the home as the place for delivery because this has been the custom for generations.” (Mother or mother in law)

##### Desire for medical treatment due to fear about child mortality and difficulties during childbirth

Participants were afraid that their child might die or have physical problems stemming from childbirth and therefore wanted medical resources such as injections and oral medicines to be available. On the other hand, they did not visit the local HCF because they did not consider lack of appetite, dizziness, fatigue, or labor pains as signs and symptoms of a disease. The participants’ perceptions of ill health were based on their experiences. For instance, they visited a HCF if they felt that their labor pains were too strong or the bleeding was excessive as they understood these experiences as manifestations of an abnormal delivery:

“I want women to deliver in a hospital because it is very convenient: there is a health care provider on hand to help, mothers can readily get an injection since medicine is available and will therefore recover more quickly and have a safe delivery.” (Village head)

“I want to deliver at a HCF because I’m afraid of experiencing difficulty during delivery and maybe dying. I don’t want to die because of a difficult delivery.” (Woman)

##### Religious beliefs about childbirth

Most ethnic minorities in this area believe in “*sadsana-phee*,” an indigenous animist belief system which is prevalent in Lao. Participants reported experiencing the presence of spirits or “*phee*” when they visited a health center and prayed to the *phee* whenever they experienced difficulty during delivery. The participant stated:

“In 2003 some women had difficulties in delivery. In such cases, a traditional healer is called to assist the delivery. The delivery lasted for 2 days in some cases. After the traditional healer prayed, the mothers delivered immediately. In 2004 there were two mothers who had difficulties during delivery and experienced pain for three days until delivery. The traditional healer assisted the delivery by praying, a process that took about 30 minutes until the women safely delivered.” (Village head)

#### Traditional health concept: *sabaai*

“Traditional health concept: *sabaai*” consisted of three subcategories including “*sabaai* as healthy,” “*sabaai* as easy,” and “*sabaai* as comfortable.” The study participants frequently mentioned *sabaai* as a determinant in their choice of the place of delivery. *Sabaai* is an expression of well-being in Lao. It is a unique Laotian concept which combines the meanings of “healthy,” “easy,” and “comfortable,” with the nuances depending on the context. This word was used to describe both home-based and facility-based delivery.

##### *Sabaai* as healthy

Participants used *sabaai* to describe the state of a woman in good health, who can therefore opt for a home-based delivery. This perception of good health meant that pregnant women who had *sabaai* did not need medical intervention. The participants explained:

“I was healthy (sabaai) so I was able to deliver at home.” (Woman)

“I delivered at home because it was easy and I wasn’t sick (sabaai).” (Woman)

##### *Sabaai* as easy

The Laotian language has a word meaning “easy” apart from *sabaai*. While *sabaai* also denotes “ease,” it has specific associations with the ease of delivery experienced either at home or at a HFC. The participant said:

“The delivery was so easy (sabaai)! I didn’t feel sick during the delivery and the baby popped out quickly.” (Woman)

##### *Sabaai* as comfortable

The study participants used *sabaai* to express comfort in both facility-based and home-based settings. At home, pregnant women experienced *sabaai* because they were giving birth in customary surroundings and family members were on hand to assist. On the other hand, some participants also reported experiencing *sabaai* while delivering at a HCF because of the cleanliness of the premises, the availability of staff ready to assist with the delivery, and the availability of medicines. The participants explained:

“Delivery at home is comfortable (sabaai) because this has been our custom for generations.” (Husband)

“For the next delivery, I want to go to a hospital. It is comfortable (sabaai) there and I can get assistance from the health care staff.” (Woman)

#### Perceptions of health care facilities and staff

“Perceptions of health care facilities and staff” consisted of two subcategories including “care at health care facilities” and “service from health care staff.”

##### Care at health care facilities

Many women reported that delivery at a HCF was *sabaai*. For them, *sabaai* meant that health care staff were on hand to attend to their delivery and that they had a good impression of the care provided. One woman said:

“Next time, I want to deliver at a hospital because it is clean and the health care staff are there to take care of us.” (Woman)

There were also women who wanted the health care staff to give them more attention during their stay in the facility. One participant said:

“I think the health care staff should have visited me more often while I was there.” (Woman)

##### Service from health care staff

The health care staff conducted outreach activities in villages including vaccinations and ANC. Many participants reported that the health care staff were “good” or kind. However, one woman expressed dissatisfaction with the attitude of a health care staff. She said:

“We want the health care staff to welcome the patient more kindly because in the past they gave good service only to the people they knew and didn’t talk much to those they didn’t know.” (Woman)

One Phouthai woman, a teacher who delivered at a HCF, said some peoples were afraid to have contact with the health care staff. However, other participants did not mention having this experience. The Phouthai woman said:

“Some people do not want to go to hospital because they are afraid to see the health care staff. These people just have to put up with giving birth at home.” (Woman)

#### Previous pregnancy and childbirth experience

“Previous pregnancy and childbirth experience” consisted of two subcategories including “previous childbirth experience” and “experience of ANC.”

##### Previous childbirth experience

The participants who experienced difficulty during childbirth or the death of their infant a few days after delivery were naturally afraid of repeating this experience. Such experiences promoted the popularity of facility-based delivery. On the other hand, participants who did not experience any problems in their previous delivery opted to deliver at home. One participant mentioned:

“I delivered at a HCF twice (third and fourth child). I made the decision to deliver my third child at a HCF during the fifth month of my pregnancy. I wanted to deliver at a HCF again before getting pregnant with my fourth child because I think the HCF is nice and clean.” (Woman)

##### Experience of ANC

The experience of ANC also influenced mothers’ choice of place of delivery. A woman who received ANC said that she received health checkups for herself and her fetus and health education on topics such as avoiding hard work and consuming the right kinds of food while pregnant. The health care staff likewise recommended making regular use of ANC. Another woman reported that a health care volunteer noticed that her abdomen was abnormally swollen. As a result, the woman decided to receive ANC at a HCF regularly following the advice of the health care staff.

Many women attended ANC sessions and chose to deliver at a HCF if they felt ill. On the other hand, some women attended ANC sessions to ensure a safe delivery even if they were not experiencing any health problems. One participant said:

“I received ANC four times because I felt dizzy. I got medicine from a HCF for ten consecutive days. Afterwards I got better. During the pregnancy there was no bleeding from my vagina.” (Woman)

#### Mode of available transportation

“Mode of available transportation” had one subcategory, “transportation to health care facility.”

##### Transportation to health care facility

Transportation to HCF influenced the choice of the place of delivery. Of the respondents interviewed, only one had a car and 49 participants had motorbikes. Some participants had to request transportation from relatives, neighbors, or a HCF due to the difficulty of transporting women experiencing labor pains by motorbike. The participants said:

“We want relatives to take care of us and help transport us to the hospital.” (Husband 40)

“I want transportation provided by my relatives and HCF.” (Husband)

Most participants answered that it was usually the husband’s responsibility to take their wife to the HCF, often by motorbike, whenever she feels ill. However, one woman reported going to a HCF alone by riding a small, local bus called *Songthaew* to minimize the cost of transportation.

#### Financial burden of childbirth at health care facility

“Financial burden of childbirth at health care facility” consisted of three subcategories including “cost of transportation to the health care facility,” “indirect cost of childbirth at a health care facility,” and “hidden cost of childbirth at a health care facility.”

##### Cost of transportation to a health care facility

Some of the participants needed to rent a vehicle to go to a HCF when they experienced labor pains because they had no car or motorbike. They reported having to pay not only the car rental fee but also the fuel costs. The cost of transportation to a HCF constituted one of the financial burdens of a facility-based delivery.

##### Indirect cost of childbirth at a health care facility

Many of the women who delivered at a HCF had to shoulder the cost of plastic sheets, cover sheets for their baby, and plastic bags for the placenta. A participant said:

“I had to buy everything by myself such as the plastic sheet, the cover sheet for my baby, and the plastic bag for the placenta. The total cost was 30,000 kips.” (Woman)

One participant reported that a health worker ordered her to buy a new cover sheet for the baby because the one she had brought with her was too rough for the baby. When the health care staff were interviewed about this, they mentioned that villagers were not required to buy supplies if they were unable to pay for them, indicating some inconsistency in the responses made by the health care staff and the participant. One participant stated:

“(When you had your delivery in the hospital, did you pay for a plastic bag for the placenta or for a baby cover sheet?) I did not buy anything. They gave it to us for free.” (Woman)

##### Hidden cost of childbirth at a health care facility

Hidden cost of childbirth at a HCF pertains to an unofficial payment called *Namchay*, given to the delivery attendant as a token of gratitude. *Namchay* is not enforced or requested by the health care staff, but women and their families feel obliged to show their gratitude for attendance at delivery due to a traditional belief, *Yanbaab*, that people sin if they fail to pay those who help them. A participant stated:

“The reason for giving an incentive (Namchay) to the health care staff is that we are afraid of sinning and our child would be tainted by the sin as well.” (Woman)

The price of *namchay* ranges from 20,000 kip to 100,000 kip. In one case, the payment was as high as 100,000 kip to 200,000 kip (12 US dollar to 23 US dollar), almost equal to the price of renting a car. The study participants felt that they had to pay this as an incentive to the health care staff even if they did not have the financial resources. The women knew that they did not need to pay for their delivery at the HCF and could visit the facility whenever they needed.

#### Family and community context

“Family and community context” consisted of two subcategories: “personal links with others” and “decision-making by the family.”

##### Personal links with others

The pregnant women and their relatives usually made decisions regarding the place of delivery. One woman received support from many relatives during and after her delivery. On the other hand, another woman did not expect any support from anyone except her husband. She said:

“No relatives gave us advice about dealing with labor pains when I had my first child. Nobody told me that we could go to a hospital for ANC so we didn’t. We received ANC when the health care workers visited our village. I helped myself (during my delivery) because my husband had a headache and was coughing.” (Woman)

##### Decision-making by the family

Many women chose the place of delivery after consultation with their husband. In addition, they also considered their own health when choosing a place. However, some participants reported that it was their mother or mother-in-law who decided the place of delivery. In one case, the woman’s mother-in-law and husband allowed her to deliver only at home. It was assumed that the woman did not have the right to decide for herself. One husband said:

“If my wife does not have any health problem I would allow her only to deliver at home because this has been our custom for generations. An easy delivery, and then we drink hot water and all is fine.” (Husband)

#### Institutional context

“Institutional context” consisted of two subcategories including “difficulty preparing for facility-based delivery” and “language difficulties.”

##### Difficulty preparing facility-based delivery

The participants noted experiencing difficulties related to preparing for facility-based delivery. The patients and their families had to prepare food for the period of hospitalization because it was unavailable at the HCF. In addition, they also needed to prepare food for the patients’ family and the cost of transportation. Participants reported feeling that it was easier to prepare for delivery at home and that the financial burden would be smaller.

##### Language difficulty

Participants also reported experiencing language difficulties at the HCF because their ethnicity differed from that of the health workers. Almost all participants were members of the Tree or Makong tribe. Also, most of the women did not attend school, and communicating in Lao was therefore difficult for them.

#### Government policy on delivery

“Government policy on delivery” had one subcategory, “free delivery policy at health care facilities.”

##### Free delivery policy at health care facilities

The national health care policy of Lao PDR ensures that mothers can receive assistance for their delivery at designated HCF free of charge. This policy was implemented to promote facility-based delivery. Most women who gave birth at home and their families were familiar with this policy. One participant emphasized:

“Before, we did not know (about the free delivery) and were afraid of spending money so we avoided going to a hospital. But we now know about it and we will go to hospital. We are not afraid anymore.” (Woman)

The village head and some of the women and the husbands, on the other hand, did not know that this service was free, indicating that the information had not been fully disseminated in the study area. One village head stated:

“The government should cover all the costs for delivery not only at the HCF but also at the district hospital in case the HCF transfers the patient to the district hospital.” (Village head)

## Discussion

This study identified nine categories influencing the choice of facility-based delivery including “perception of childbirth,” “traditional health concept: *sabaai*,” “perception of health care facilities and staff,” “previous pregnancy and childbirth experience,” “mode of available transportation,” “financial burden of childbirth at health care facility,” “family and community context,” “institutional context,” and “government policy on delivery.”

### Positive factors influencing choice of facility-based delivery

Four categories, namely, “perception of health care facilities and staff,” “previous pregnancy and childbirth experience,” “mode of available transportation,” and “government policy on delivery” positively influenced the choice of facility-based delivery.

“Perception of health care facilities and staff” did not demonstrate any negative influence on facility-based delivery, although previous studies reported embarrassment or discrimination at a HCF [11, 15, 16]. The absence of such an influence in the present study might be due to the efforts to improve health care in Xepon in the last 10 years. “Previous pregnancy and childbirth experience” such as strong labor pains, difficult delivery, infant death, and use of ANC had positive influences on respondents’ perceptions of facility-based delivery. Women who had transportation went to a HCF for delivery. “Government policy on delivery,” such as the free delivery policy at HCFs, promoted the choice of facility-based delivery. However, the participants did not understand the policy well possibly due to language difficulties and lack of communication between the members of the community and health workers.

### Negative factors influencing choice of facility-based delivery

The remaining categories negatively influence the perception of facility-based delivery. “Perception of childbirth including childbirth as a natural event rather than as a disease” was a barrier to facility-based delivery, as corroborated by previous studies [11, 17, 18].

“Traditional health concept: *sabaai*” had both positive and negative influences on the choice of facility-based delivery. Women who delivered at a HCF felt *sabaai* but most of the participants felt *sabaai* regarding home-based delivery. The choice of facility-based delivery might receive a boost if people understood that the advantages of facility-based delivery could provide more *sabaai* than home-based delivery.

“Financial burden of childbirth at health care facility” such as transportation expenses and the “indirect and hidden costs of childbirth at a health care facility” negatively influenced the choice of facility-based delivery. This result was also corroborated by previous studies [10, 11, 19, 20]. While as previously mentioned delivery at HCF is free under the current Laotian health care policy, this study disclosed indirect costs such as food, cover sheets, plastic bags, and unofficial payments to the delivery attendant. A previous study showed that health policies were not functioning well because health care workers failed to understand and communicate this policy well [21, 22].

“Family decision-making” had both positive and negative influences on the choice of facility-based delivery. This result was also corroborated by previous studies [10, 11, 23, 24]. The husband or another family member chose facility-based delivery if they thought that the woman required it; otherwise, home-based delivery was chosen. Husbands are reportedly responsible for providing their wife with emotional, material, and informational support during delivery at a HCF [17]. Aguiar [25] showed that men’s presence at ANC sessions was associated with increased institutional delivery and skilled birth attendance, suggesting that better strategies are needed to expand male involvement in order to increase facility-based deliveries.

“Institutional context” including “difficulty preparing for facility-based delivery” and “language difficulty” negatively influenced the choice of facility-based delivery. The difficulty experienced by women and their families in preparing for a facility-based delivery was corroborated by previous studies [10, 11]. However, language difficulties were not reported in the same studies. Health care workers need to understand the potential for language difficulties and provide support accordingly.

## Recommendations [2]

**Fig. 1:**
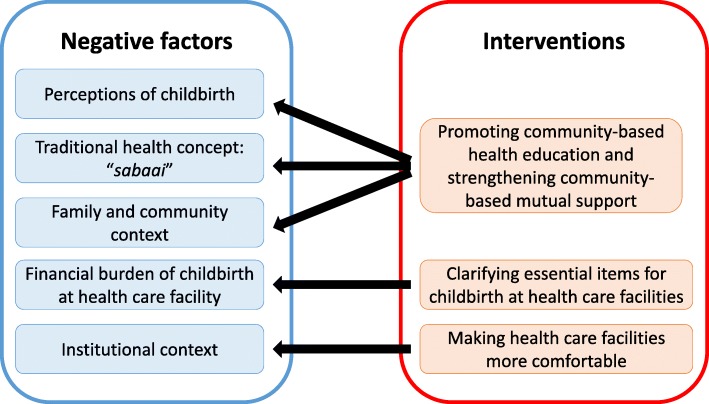
Intervention to mitigate negative factors influencing the choice of facility-based delivery

Based on our findings, the following intervention strategies are recommended to promote facility-based delivery in the rural districts of Lao PDR (Fig. 1). First, women’s groups should be supported in order to promote community-based health education and strengthen community-based mutual support. Prost [26] showed that health education provided by women’s groups was a cost-effective strategy for reducing MMR and IMR in developing countries. Prost also reported that community-based mutual support improved access to HCF and resulted in the establishment of a fund to pay for medical services. Positive changes in the behavior of women and their community will likely result if women’s groups lead the effort to educate the public about facility-based deliveries and the risks of childbirth.

Second, it is necessary to clarify the resources required for delivery at a HCF and to inform pregnant women, their family, VHVs, and the village heads of the availability of these resources. A women’s group can take an active role in the development and dissemination of this information in their respective community. Despite the official policy of free facility-based delivery in Lao PDR, indirect costs are sometimes incurred by women and their families when preparing for delivery at a HCF. If a list of essential items for delivery is provided, pregnant women can better prepare for delivery through receiving more specific support from their relatives or women’s groups.

Third, HCF should be made more comfortable. The quality of HCF in Xepon district was targeted by the Laotian government for long-term improvement. As a result of this effort, people’s awareness of HCF has grown. Nonetheless, further improvements such as providing more comfortable care to patients should be made and will reinforce the community’s positive perceptions of this vital resource.

This study demonstrated that the difficulties experienced during delivery at a HCF including language problems for ethnic minority members, the unavailability of food, and the separation of women from their family contributed to negative perceptions of facility-based delivery. The concept of “*sabaai*” is an important part of the Laotian culture. Incorporating this concept into the public health care system will undoubtedly improve people’s perception of facility-based delivery.

## Limitations

The above findings should be taken in the light of the following limitations of the study: The presence of the villagers during the interview might have introduced a social desirability bias because of the familiarity between the interviewers and participant. However, this potential source of bias was minimized by explaining to the informant the confidentiality of the information that will be collected from the study. The limited number of interviewers who speak both Laotian and the ethnic minority language was a particular challenge in this study.

Because of the high dropout rate in the formal education, most of the study participants could understand only ethnic minority language. However, this was minimized by the presence of the villagers who helped during the interview.

## Conclusion

Our study demonstrated that five major factors negatively influenced the choice of facility-based delivery: (1) perception of childbirth, (2) preference for *sabaai* (a condition of health, ease, and comfort), (3) financial burden, (4) family decision-making, and (5) institutional context.

To promote facility-based delivery in the rural district of Xepon, three strategies are recommended: (1) promoting community-based health education involving women and strengthening community-based mutual support, (2) clarifying items essential for delivery at HCFs, and (3) making HCFs more comfortable in terms of “*sabaai*.”

## Data Availability

Raw data may be obtained from the corresponding author upon request.

## Abbreviations

ANC: Antenatal care
FGD: Focus group discussion
HCF: Health care facility
IDI: In-depth interview
IMR: Infant mortality rate
Lao PDR: Lao People’s Democratic Republic
MMR: Maternal mortality ratio
U5MR: Under 5 mortality rate
VHV: Village health volunteer
